# Coupling of Immobilized Photosynthetic Bacteria with a Graphene Oxides/PSF Composite Membrane for Textile Wastewater Treatment: Biodegradation Performance and Membrane Anti-Fouling Behavior

**DOI:** 10.3390/membranes11030226

**Published:** 2021-03-22

**Authors:** Jing Cheng, Xiaofeng Wu, Binbin Jin, Chenchen Zhang, Rongwei Zheng, Lei Qin

**Affiliations:** 1 Department of Hydraulic Engineering, Zhejiang Tongji Vocational College of Science and Technology, Hangzhou 311231, China; feijing1987@126.com (J.C.); zhangcc1988@foxmail.com (C.Z.); zjzrw0910@126.com (R.Z.); 2 Yiwu Academy of Science and Technology, Zhejiang University of Technology, Jinhua 322000, China; zjutywyjy@163.com; 3 College of Water Conservancy and Hydropower Engineering, HoHai University, Nanjing 210098, China; 4 Institute of Oceanic and Environmental Chemical Engineering, Zhejiang University of Technology, Hangzhou 310014, China

**Keywords:** membrane modification, GO nanosheets, immobilized cells, membrane fouling, wastewater treatment

## Abstract

The membrane bioreactor (MBR), as one of the promising technologies, has been widely applied for treatments of wastewater. However, serious membrane fouling and low microbial activity have been reported as major problems hindering the development of the MBR. To overcome these drawbacks, we intend to improve the MBR process in the view of membrane surface modification and efficient granular bacteria cultivation. In the present study, immobilized photosynthetic bacteria integration with graphene oxide (GO)/polysulfone (PSF) composite membrane separation (IPMBR) was first applied for textile wastewater treatment. Due to the high activity of immobilized cells, the IPMBR system exhibited higher efficiency on the removal of color, ammonia–nitrogen, and chemical oxygen demand than the conventional MBR system. In comparison with a pure PSF membrane, GO/PSF composite membrane presented the higher hydrophilicity (water contact angles of 62.9°) and more attractive permeability (178.5 L/m^2^h) by reducing the adhesion of hydrophobic foulants. During the whole operation, the immobilized photobioreactor exhibited approximately seven times higher membrane permeability that that of the conventional MBR. Meanwhile, the effect of the structure and character of immobilized photosynthetic bacteria on the membrane fouling reduction was investigated in detail. The change of extracellular polymeric substance concentration, settleability and particle size of flocs was very beneficial to alleviate membrane fouling. As a result, this research will open a new avenue for developing efficient and anti-fouling MBR technology in the future.

## 1. Introduction

Along with rapid growth of social economy and the blooming process of industrialization, textile printing and dyeing wastewater generated in China has reached one million tons every year, which often pollutes groundwater and poses a more significant risk to human health [1,2]. However, treatment of dye wastewater is a very difficult task, since it still has properties of high color, heavy poisonousness and is non-biodegradable even in a very low concentration [3,4]. Therefore, there is an urgent need to develop new technologies for textile wastewater advanced treatments.

In recent years, diverse treatments processes, for example, coagulation, advanced chemical oxidation, adsorption, and biodegradation treatment, have been applied to solve the dye wastewater problem [5,6]. However, high cost, low efficiency, secondary pollution, and extra additive requirements are some troubling disadvantages to these methods. To overcome those disadvantages and problems, there is an efficient option, biological treatment, the most used method in practice over the world because of its simplicity and low cost [7,8]. Compared with traditional biological methods (including activated sludge and the sequencing batch reactor), the submerged membrane bioreactor (SMBR) has great potential as a most promising biological treatment technology [9,10,11,12]. Nevertheless, the previous research has demonstrated that the conventional aerobic MBR is not considered a saving and feasible alternative owing to the low biodegradability of reactive soluble dyes [13,14]. Moreover, membrane fouling due to the poor settling properties and small size of the microbe cell are always considered as main problems limiting the wide application of the SMBR [15]. Over past decades, lots of strategies have been developed to alleviate membrane fouling including the membrane surface modification, operating conditions optimization, and design of the combined systems [16,17,18]. However, it is worth noting that the structure and characteristics of flocs in the bioreactor are important for membrane fouling reduction. Therefore, the development of efficient MBR technology will become the key to solving environmental pollution problems.

In order to address these difficulties, previous studies have paid more attention for screening and cultivating highly efficient strains rather than the activated sludge for treating refractory organic wastewater [19]. Results showed that using photosynthetic bacteria (PSB) is a promising strategy for improving the bio-treatment of reactive dye in the biological process [20]. Meanwhile, the cultivated PSB cells are always applied for high-value nutrition, animal feed and agrochemical applications [21,22]. However, suspended PSB is easily influenced by the hydraulic condition, which is susceptible to other living creatures. Moreover, reducing membrane biofouling remains a significant challenge while operating under high biomass conditions [23]. Therefore, the immobilizing PSB within the carrier is very important, which can obviously improve their settling property, poison resistance, and tolerance [24]. As well known, microorganism immobilized technology has been used in field of environmental protection, biological production and pharmaceutical engineering [25,26]. As reported in the previous studies, the use of an immobilized carrier provides many advantages over protecting them from environmental stress/shock loading and be easily recovered, compared with suspension cultures [27]. Especially for MBR application, small-sized microorganisms entrapped within capsules were very efficient for membrane biofouling reduction [28]. In recent years, microorganisms encapsulated into spherical alginate beads have been applied in various applications owing to their high biocompatibility and simple preparation [29]. Moreover, membrane modification containing nanomaterial blending as a promising alternative has been a topic of much interest [30]. Among various nanomaterials, graphene oxide (GO) nanosheets, as a new type of two-dimensional structural material, gained more attentions in membrane modification due to their high surface area, strong hydrophilicity of oxygen-contained groups, and good compatible and mechanical properties [31]. As reported by previous studies, a polyvinylidene fluoride (PVDF) membrane blended with GO or GO/Cu_x_O nanoparticles was successfully used for wastewater treatment, which exhibited higher hydrophilicity and permeability, lower cleaning frequency, and producing longer filtration time than the pristine membrane [32,33]. Thus far, the fabrication of an antifouling membrane by blending GO nanosheets into the polymer host has been often performed; however, there have been no reports on development of an advanced membrane bioreactor (MBR) system coupled with the immobilized microbes with a modified membrane.

Based on the previous observations and our research, we intend to develop a novel immobilized PSB integrated with GO/polysulfone (PSF) composite membrane for methylene blue wastewater treatment, in which gathering of highly active PSB on chemical oxygen demand (COD) and NH_3_-N removal can be easily performed on a large scale and membrane biofouling can be effectively mitigated by the incorporation of GO nanosheets and granular bacteria. The morphology and surface properties of the prepared GO/PSF composite membrane were characterized. To compare with the original PSF membrane, the antifouling behavior of the GO/PSF composite membrane was evaluated in the immobilized photobioreactor. For comparison, an MBR with immobilized photosynthetic bacteria and a controlled MBR with suspend cells were operated in parallel for 30 days. In this work, we mainly evaluate the performance of an immobilized photosynthetic bacteria MBR (IPMBR) in terms of biomass concentration, organic contaminants removal efficiency, and membrane permeability. Moreover, the variations on the properties and microscopic structure of flocs in two MBRs, such as settleability, particle size and extracellular polymeric substance concentration (EPS) were also analyzed for a better understanding of the anti-fouling behavior.

## 2. Materials and Methods

### 2.1. Chemicals and Materials

The commercial methylene blue is available from Beta pharma Co., Ltd. (Shanghai, China). Sodium alginate (SA), polyethylene glycol, calcium chloride (CaCl_2_), sodium chloride (NaCl) and sodium hydroxide (NaOH) were bought from Sinopharm chemical Reagent Co. Ltd. (Hangzhou, China). The components of textile wastewater are shown in Table 1, showing a high organic pollutant loading (max COD of 1025 mg/L) and ammonia nitrogen (max NH_3_-N of 112 mg/L). Moreover, the pH of wastewater was varied between 6.0 and 8.0. Photosynthetic bacteria (PSB) were selected from purified and identified *Rhodopuesdomonas pulstris*, which were cultured and grown in RCVBN medium (0.1 g/L NH_4_Cl, 0.5 g/L NaCl, 0.5 g/L NaHCO_3_, 3.0 g/L CH_3_COONa·3H_2_O, 0.1 g/L MgSO_4_·7 H_2_O, 0.1 g/L CaCl_2_·2H_2_O, 0.2 g/L K_2_HPO_4_, 0.5 g/L yeast extract, and 0.5 g/L peptone) under shaking. Throughout the whole cultivation, the irradiate intensity, solution pH and temperature were altered to approximately 3000 lux, 7.5 and 25 °C, respectively.

### 2.2. Preparation of GO/PSF Composite Membrane

The GO/PSF composite membrane was prepared through the phase inversion method. Firstly, Graphene oxide (GO) nanosheets were prepared from the Nanjing Xianfeng Nanomaterials Co., Ltd. (Nanjing, China) by means of the modified Hummers method [34]. Secondly, the as-synthesized GO nanosheets (1 wt %) and post-treated polysulfone (18 wt %) were dissolved in dimethylacetamide (DMAc). Then, the mixture solution was cast onto a clean glass plate, evaporated in air and placed in deionized water coagulation bath.

### 2.3. Immobilizing of Photosynthetic Bacteria into Hydrogel

In this work, PSB cells were encapsulated into hydrogel by way of the embedding method. The pathway used to fabricate sodium alginate (SA) entrapped beads was similar to the previously reported pathways [35]. The embedding agent solution was consisted of the polyethylene glycol and sodium alginate. The cultivated photosynthetic bacteria were immersed into the embedding agent for 20 h. Depending on the saturated boric acid solution (cross-linking agent), the hydrogel macrospheres with 2–4 mm diameter was prepared with large numbers of PSB cells. Subsequently, immobilized sphere was immersed in 2% CaCl_2_ solution further fixed in the fridge, and which was then washed with sterile water and stored in 0.85% NaCl solution.

### 2.4. Characterizations

The structure and morphology of modified membranes were characterized by JEM-1200EX scanning electron microscopy (SEM, Hitachi, Tokyo, Japan). The water contact angle (CA) of the GO/PSF composite membrane was measured by using the sessile drop method (OCA50AF, Dataphysics, Filderstadt, Germany). The surface functional groups of GO nanosheets were analyzed through Fourier Transform Infrared spectroscopy (FTIR, iS50, Thermo Fisher Nicolet, Waltham, USA). X-ray diffraction (XRD, X’Pert PRO, PNAlytical, Holland) was used to investigate the crystal structure of nanosheets. Scanning electron microscopy (SEM) images of outside surface morphology for cell-encapsulated particles were performed. Living/dead cells of encapsulated hydrogel spheres were visually monitored by the inverted fluorescence microscope.

### 2.5. Experimental Setup

**Figure 1:**
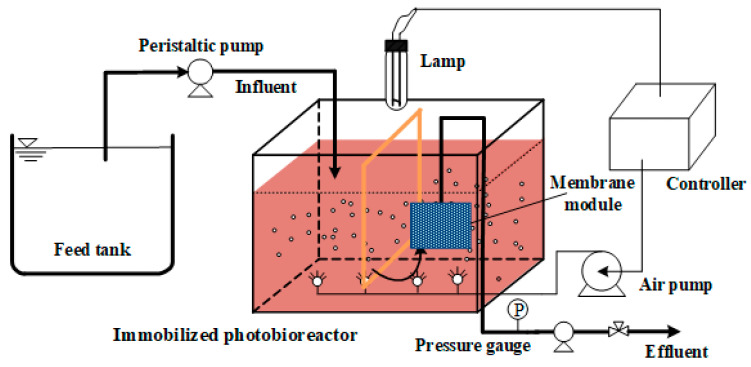
Schematic diagram of the experimental process.

The laboratory equipment used in this study is shown in Figure 1. To analyze the influence of membrane modification and cell immobilization on dye degradation and membrane fouling, two SMBRs of the same size were run in parallel under the same conditions with a working volume of 10 L, one an immobilized photosynthetic bacteria MBR (IPMBR) and another a controlled MBR (CMBR). A level sensor connected with a feeding pump was used for constant volume control. The self-made flat sheet GO/PSF composite membrane module with the effective membrane area of 0.05 m^2^ was immersed into the bioreactor. Air (1.5 m^3^/m^2^h) was continuously poured into every reactor at the same flow rate. The effluent was supplied back to the bioreactor continuously, while the SMBR system was operated in constant flow mode with 10 min inhalation and followed by 2 min relaxation. The transmembrane pressure (TMP) was continually monitored as a signal to indicate the membrane fouling. The hydraulic residence time (HRT) and solid residence time (SRT) were set at 40 h and 30 d, respectively.

### 2.6. Measurement of PSB Biomass Concentration

The concentration of PSB cells was measured by using the spectrophotometry method at the wavelength of 805 nm, being calculated as follows (Equation (1)):

(1) $$Biomass\left(g/L\right)=1.3677\times OD_{805}-2.7\times 12^{-2}\left(R^{2}=0.9987\right)$$

where *OD*_805_ is optical density of the free PSB solution; *Biomass* presents the dry weight of microbes.

The PSB biomass of hydrogel was determined through the weight difference method. A certain number of hydrogels were placed in an oven at 80 °C for 6 h to dry, and the weight of which was marked as m_1_. Additionally, the hydrogel was then heated at high temperature to volatilize bacteria, and the weight of which was marked as m_2_. The difference value of m_1_ and m_2_ is the weight of PSB encapsulated in hydrogel.

### 2.7. Calculation of Membrane Permeability and Filtration Resistance

Membrane fouling of the different integrated systems was evaluated through the variation of membrane flux over time. The membrane permeability was calculated by Equation (2) as follows:

(2) $$L_{p}\left(L/m^{2}\cdot \mathrm{bar}\right)=\frac{J_{p}}{\Delta p_{TM}}$$

where *J_p_* is the permeate flux (L/m^2^h), Δ*p_TM_* represents the transmembrane pressure (bar).

The fouled membrane was cleaned after 15 days by physical and chemical methods. The total filtration resistance (*R_t_*, m^−1^) is determined by Darcy’s law (Equation (3)):

(3) $$R_{t}=R_{m}+R_{f}=\frac{\Delta p_{TM}}{\mu \cdot J_{p}}$$

where *R_t_*, *R_m_* and *R_f_* present the total membrane filtration resistance (m^−1^), the original membrane filtration resistance (m^−1^) and the filtration resistance after fouling (m^−1^), respectively. *µ* represents the permeate viscosity (Pa^−1^·s). To evaluate membrane fouling, the surfaces of membranes before and after filtration were also examined by SEM.

### 2.8. Analytical Methods

To measure and estimate the ammonia–nitrogen (NH_3_-N), COD, mixed liquor volatile suspended solids (MLVSS) and sludge volume index (SVI), standard analytical methods were carried out [36]. The concentration of methylene blue (MB) was determined by a U-2910 digital spectrophotometer (Hitachi, Japan). The size distribution of the flocs in the MBR was determined by a Malvern Mastersizer 2000 (Malvern, Worcestershire, UK). Heat treatment was used to extract the EPS from free microbes and cells-encapsulated flocs, and the total of the EPS was calculated and regarded as total organic carbon (TOC) contents based on the sample supernatant.

## 3. Results and Discussion

### 3.1. Characterization of GO Modified Composite Membrane

**Figure 2:**
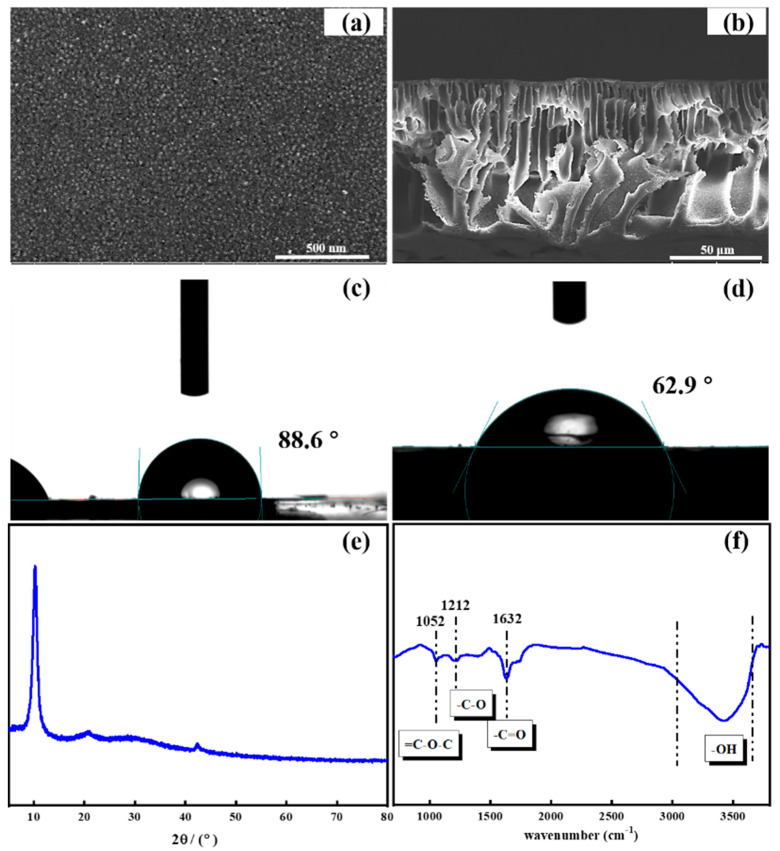
(**a**,**b**) Scanning electron microscopy (SEM) images of prepared graphene oxide (GO)–polysulfone (PSF) composite membrane; (**c**,**d**) water contact angle of original PSF and GO-PSF composite membrane; and (**e**,**f**) X-ray diffraction (XRD) and Fourier transform infrared spectroscopy (FTIR) of the prepared GO nanosheets.

The surface structure of the prepared GO/PSF composite membranes was characterized by SEM images and is shown in Figure 2a and b. As apparent from the figure, it was found that the modified GO/PSF membrane presented the rough surface owing to the exposure of blended GO nanosheets. The cross-sectional image displays that the prepared ultrafiltration membranes present a typical construction with an asymmetric finger-like porous sub-layer and a dense skin layer. By comparison, the phase inversion rate after altered after the introduction of hydrophilic GO nanocomposites, making the pores of the membrane larger so that water molecules can pass through the membrane easier. In the XRD pattern, the characteristic peak at 2θ = 10.3° belongs to GO nanosheets (Figure 2e). Figure 2f presents the FTIR spectra of prepared GO nanosheets. As apparent from the figure, the typical peak at 1632 cm^−1^ which appeared was assigned to C=O stretching vibrations of the GO nanosheet. Moreover, the band at 1052 cm^−1^ (=C-O-C) and the stretching vibration band at 1212 cm^−1^ (-C-O) were obviously shown in the curve of the GO nanosheets [37].

### 3.2. Hydrophilicity and Filtration Performance of GO Modified Membrane

The water contact angle was measured to investigate the surface hydrophilicity of original PSF and GO/PSF composite membrane. As illustrated in Figure 2c,d, the water contact angle of GO/PSF composite membrane reduced from 88.6 to 62.9°. It indicated that the surface hydrophilicity of membrane was improved by the incorporation of GO nanosheets, and thus significantly enhanced the membrane permeability. The details in the pure water flux and bovine serum albumin (BSA) rejection of the PSF membrane before and after GO modification was investigated. It was found that the pure water permeability of original PSF membrane was 63.3 L/m^2^·h. After GO blending, the increase in the water flux to 178.5 L/m^2^·h was observed for the modified membrane, which is approximately 2.82 times higher than that of pure PSF membrane. Similarly with the reported GO modified membranes, the prepared composite membrane also exhibited excellent flux, which demonstrated that introducing hydrophilic GO nanosheets into the membrane matrix can the increase water transport rate [37,38]. In comparison with the PSF membrane (90.5%), the GO-blended composite membrane possessed the higher rejection (95.1%). The enhanced rejection of the modified membrane improved the concentration of microbes for wastewater treatment.

### 3.3. Preparation and Characterization of Immobilized Particles

**Figure 3:**
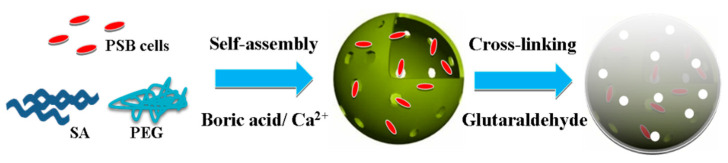
Schematic diagram of preparation of the photosynthetic bacteria (PSB)-encapsulated hydrogel macrosphere.

**Figure 4:**
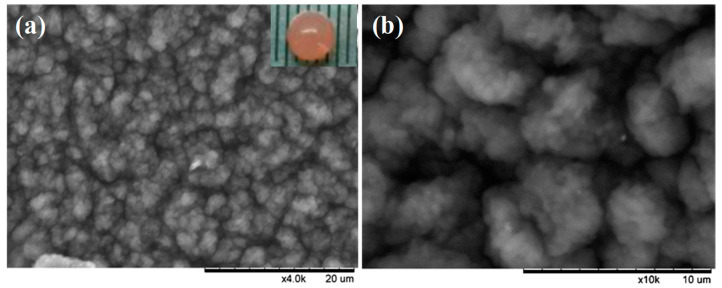
SEM images and photograph of the PSB-encapsulated hydrogel macrosphere.

As illustrated in Figure 3, the encapsulation of PSB cells into a porous polymer hydrogel involved two procedures: (1) fast self-assembly of the polymeric hydrogel containing PSB cells, sodium alginate (SA), and polyethylene glycol (PEG) through the coordination of Ca^2+^; (2) strong cross-linking between the hydrogel and glutaraldehyde. The high biocompatibility and large cavity of the polymeric hydrogel provides a favorable atmosphere for PSB cells growth. Meanwhile, the cross-linked layer on the surface of macrocapsule effectively reduced the leakage of cells into solution. The surface morphology and microstructure of PSB-encapsulated macrocapsules was characterized by SEM. As shown in Figure 4a,b, the surface of encapsulated particles was rough, which may be attributed to the immobilization of some microorganisms into the interior cavity of the hydrogel. The diameter of globular macrocapsules was ranged from 4.0 to 5.0 mm. In terms of surface color, it was found that the polymer hydrogel was transparent, which had a positive effect on the light utilization of the encapsulated microbe’s cell.

**Figure 5:**
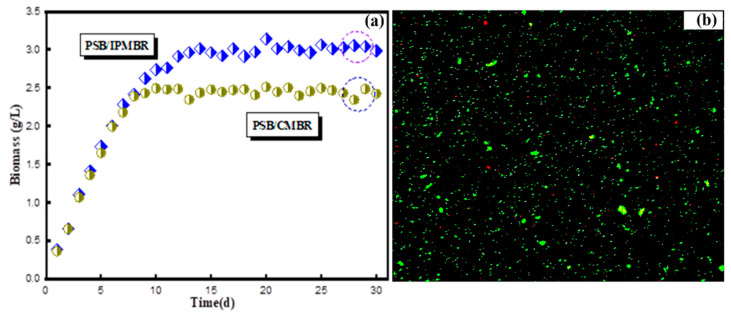
(**a**) Biomass productivity of the suspended and encapsulated system with operation time increasing. (**b**) Inverted fluorescence microscope photograph of the PSB-encapsulated hydrogel macrosphere.

Biomass production of free and encapsulated PSB cells with operation time increasing was monitored and measured, and the results of volumetric biomass content for two different CMBR and IPMBR systems are presented in Figure 5a. In the initial stage (3d), the free PSB concentration presented a rapid upward trend, and the biomass of the MBR system increased from 0.37 to 1.07 g/L. After 10 days, the content of biomass reached 2.49 g/L, maintaining a constant state for a long time. In comparison with the MBR, the content of encapsulated PSB cells was as high as 3.07 g/L, indicating the enhancement of PSB biomass production in the IPMBR. On average, approximately 23.3% of biomass yields for the IPMBR system were higher than the CMBR. By comparison with the previous studies, the enhanced biomass maybe due to fact that the micro-aerobic environment of the interior cavity allowed the encapsulated PSB cells to obtain more energy to improve organics’ assimilation ability and maintain a high growth rate [39]. The inverted fluorescence microscope photograph also highlighted that many living cells were observed inside the cavity of the porous polymer hydrogel, while only a few dead cells (red) were shown (Figure 5b). However, it was found that the leakage of cells (2.65 g/L) occurred during the operation. Therefore, the structural stability and mechanical strength of the polymeric macrospheres should be further strengthened in future study.

### 3.4. Performance of the Integrated System

**Figure 6:**
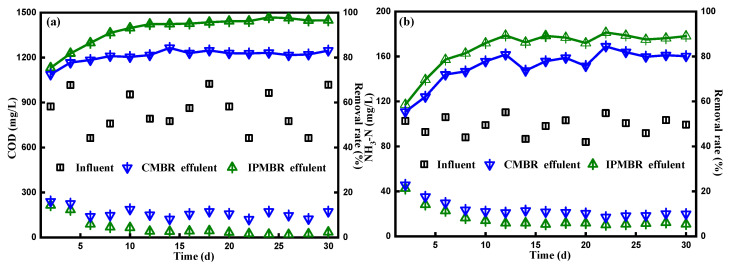
(**a**) Variation of the concentration of chemical oxygen demand (COD) and corresponding removal efficiency and (**b**) variation of the content and removal efficiency of NH_3_-N in different PSB–membrane bioreactors (MBRs) systems.

The true color, NH_3_-N of influent and effluent, the average COD and removal efficiency corresponding to the overall continuous process are presented in Table 2. As illustrated in Figure 6a, it was found that, during the entire operation period, the COD of textile influent floated between 1025 and 662 mg/L. In the initial stage, the effluent COD fluctuated between 240 and 225 mg/L. A total of 6 days later, the organic contaminants’ removal efficiencies of the photo-bioreactor were enhanced steadily and afterwards maintained the removal rate up to 82.0% after bacterial adaptation in the PSB–CMBR system. In a sharp comparison, the COD concentration decreasing drastically from 150 mg/L to lower than 40 mg/L was observed in the IPMBR system, which maintained an average removal rate up to 92.6%, much higher than that in the CMBR system. The tendency of COD removal performance was analogous to that of biomass production. It was suggested that the encapsulation strategy could improve the organic pollutants’ degradation of microbes in the MBR. Figure 6b shows the ammonia nitrogen concentration and its related removal rate in the the IPMBR and CMBR. As mentioned before, the removal rate of NH_3_-N in the CMBR was quite low at the start of operation, and the average rate was around 55.4%. After several days, an apparent reduction in the content of NH_3_-N for the CMBR effluent from 45.7 to 23.8 mg/L was observed, presenting ammonia nitrogen removal with an average efficiency of 75.7%. As shown in the figure, the NH_3_-N removal efficiency was greatly enhanced in the IPMBR with efficiency of 90.7%, which is in accordance with the trend of organic pollutants’ removal performance. At the same time, a similar trend was also found for the color removal, the efficiency of which for the IPMBR (92.3%) was much higher than that for the CMBR (74.8%). Compared with the CMBR, the higher biodegradation performance of the IPMBR was mainly attributed to the fact that the immobilization strategy created the confined environment for PSB harvesting and protected living cells away from physical/chemical stress [40,41]. This reveals well that immobilizing of photosynthetic bacteria played positive roles on the dye wastewater treatment.

### 3.5. Comparison of Membrane Permeability on Different Integrated Systems

**Figure 7:**
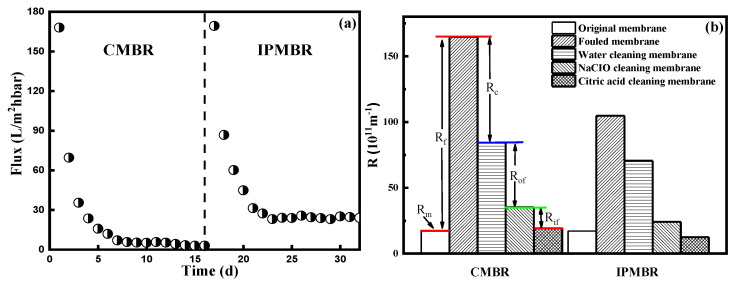
(**a**) Time-variation of permeability on the membrane during the controlled MBR (CMBR) and immobilized photosynthetic bacteria MBR (IPMBR) operation and (**b**) filtration resistances calculated during the filtration with different PSB-MBRs.

Membrane permeability in the CMBR and IPMBR systems with the time variation during the operation periods is shown in Figure 7a. Evidently, the initial permeability of the two reactors decreased rapidly. In the CMBR, the permeability of the membrane decreased from initial 168.1 to 69.6 L/m^2^·h·bar during 24 h operation. However, the membrane flux was constant about 3.7 L/m^2^·h·bar after 9 days. In the IPMBR, the membrane permeability was 169.3 L/m^2^·h·bar, well above the CMBR in the corresponding period, and the steady-state permeability was 24.1 L/m^2^·h·bar. It demonstrated that the addition of immobilized photosynthetic bacteria significantly inhibits the membrane fouling.

**Figure 8:**
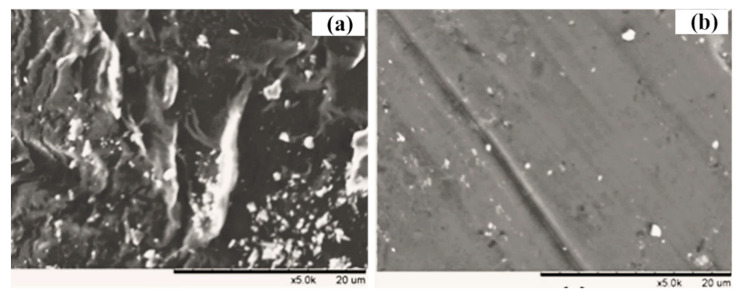
SEM images of fouled membrane in the CMBR (**a**) and in the IPMBR (**b**).

To further explore the main reasons of fouling behavior in two integrated systems, the membrane filtration resistances were calculated (Figure 7b). By comparison, obviously the membrane resistance (R_m_) was the minority of overall resistance (10.5% and 16.4% for the two MBR systems), but higher resistance due to the formation of a cake layer was observed during filtering [42,43]. In comparison with the CMBR (48.8%), it was found that the significant reduction in filtration resistance (Rc, 32.7%) in the cake layer was shown in the IPMBR system. Meanwhile, SEM images of fouled membranes were characterized to analyze the deposition of flocs on the surface (Figure 8). After operation in the CMBR later, nearly all the surfaces of the membrane were fully covered by cake layer densely. However, evidently the deposition of fewer particles on the membrane surface during the IPMBR process was observed compared with the CMBR. The observation illustrated well that the cell encapsulation strategy played vital roles in protecting the deposition of cake layer on the membrane surface. Moreover, it was demonstrated that large numbers of PSB cells were immobilized into porous macrospheres, which could be beneficial to enhancing the performance of the MBR in treating wastewater. It was noted that the filtration capacity of the membrane was closely related to the characteristics of sludge flocs in the bioreactor [44,45]. Therefore, in order to understand what influences immobilized photosynthetic bacteria in terms of membrane fouling, the various characteristics of the immobilized microorganism were investigated in detail.

### 3.6. Characteristics of Sludge Flocs in Integrated System

**Figure 9:**
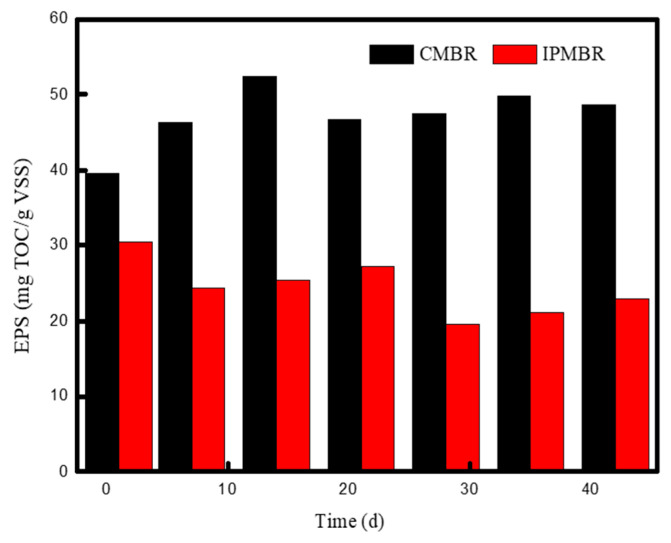
Variation of extracellular polymeric substance concentrations (EPS) of microorganism with operation time in different MBRs.

Extracellular polymeric substance (EPS) is usually considered to be the main structural component of the sludge flocs, whose characteristics and concentration played a significant role in the membrane permeability [46]. In this study, the role of cell immobilizing on the flocs’ properties was investigated based on the bound EPS (bEPS) concentration. As is shown in Figure 9, the content of bEPS in the bioreactor varied significantly with operation time in the two MBRs. For the CMBR system, the quantity of EPS increased from 39.5 to 52.4 mg-TOC/g-MLVSS in the initial 14 days. However, the concentration of EPS was stabilized, maintaining around 47.0 mg-TOC/g-MLVSS after 20 days. The concentration of bEPS in the IPMBR was lower than that in the CMBR, and the value range of which was varied from 20 to 30 mg-TOC/g-MLVSS. The obvious decrease in bound EPS concentration may be attributed to large amount of of polymeric substances adsorbed on the surface of porous supports. Moreover, the shell of the hydrogel macrospheres prevented the release of metabolic polymeric substance into mixed liquid. As previous studies reported, there was no significant linear relationship between bEPS and TMP, and the low concentration of bEPS was conductive to the decline in TMP and enhancement of membrane filtration [47].

As is well known, the influence of flocs’ size on the membrane permeability is usually explored in the MBR system [48,49]. As isapparent from the figure, it was found that large numbers of small photosynthetic bacteria were aggregated into the immobilized polymer hydrogel, and the diameter of the sphere was about 3.2 mm, much larger than that of conventional PSB bacteria (0.5–2 μm) of the CMBR system. Here is the Carman–Kozeny equation (Equation (4)), providing vital information of the specific resistance (α) of the cake layer relating closely to the particle size, and it is inversely proportional to square of the particle diameter:

(4) $$\alpha =\frac{180{\left(1-\varepsilon \right)}^{2}}{\rho_{p}d_{p}^{2}\varepsilon^{3}}$$

where *ε* is the cake porosity, *d_p_* is the particle size (m), and *ρ_p_* presents the particle density (kg/m^3^). The results confirmed that the obvious increase in the particles size of small microorganism cell significantly improve the permeability of the IPMBR system.

**Figure 10:**
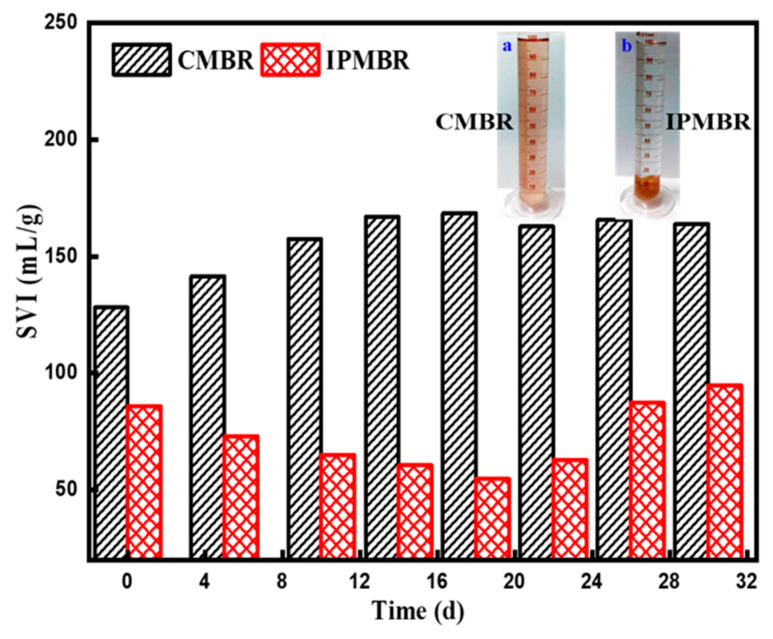
Variation of sludge volume index (SVI) of microorganisms with operation time in different MBRs and photographs of the microorganism in the CMBR (**a**) and IPMBR (**b**) systems.

In general, the aggregate structure of primary flocs plays a vital role in the physicochemical properties of flocs including flocculation capacity and sediment ability. SVI is often applied to estimate the settleability and compressibility of flocs. Better settleability can enhance the porosity of cake layer, thus increasing the permeability of the membrane [50]. Figure 10 presents the variations of SVI with increasing of operation time in the CMBR and IPMBR. Different traditional activated sludge, the settleability of PSB cells in the bioreactor is always poor, which was attributed to the rare presence of microbe-attached particles. It was found that the microbes of the CMBR held the poor settleability with SVI of 128.14 mL/g in the initial stage. With increasing operation time, a slight increase in SVI to 168 mL/g was observed for the CMBR. As reported by previous studies, the high EPS concentration had a passive impact on flocs’ compressibility and settleability [51,52]. Therefore, it suggested that the weakened settleability of PSB cells in the CMBR can be due to more negatively charged substances adsorbed on surface of living cells. In sharp contrast, a quick reduction in SVI from 85.7 to 60.5 mL/g was observed in the initial stage of operation in the IPMBR system. When operated at 18 days, the SVI was descended to 54.7 mL/g, substantially lower than that of microbes in the CMBR systems. However, when the operation time was further prolonged, the SVI of the IPMBR was obviously increased owing to the leakage of PSB cells from the polymeric hydrogel. Moreover, the photograph of the mixed liquid also evidently presented an influence of immobilizing on the settleability and morphological feature of microbes compared with the CMBR. This reveals that the compressibility and settleability were significantly improved in the IPMBR system. The above results well demonstrate that the encapsulation strategy played important roles in the compressibility and settleability of microbes.

## 4. Conclusions

In this study, a GO/PSF composite membrane was integrated with immobilized a PSB bioreactor for wastewater advanced treatment. A GO blended membrane with enhanced hydrophilicity and antifouling property was successfully applied for high-active PSB strain harvesting. The results show that the novel combined process on COD, color, and NH_3_-N removal were excellent and stable with averages > 88%. Owing to introduction of GO nanosheets and granular bacteria, the IPMBR process exhibited approximately seven times higher membrane permeability than the conventional MBR system. The decrease in EPS content, the improvement of compressibility and settleability, and the increase in particle size benefited membrane reduction. Therefore, this work will bring new insight for the development of efficient and anti-fouling MBR technology for various applications.

## Figures and Tables

**Table 1:** Synthetic textile wastewater characterization.

Composition	Concentration (mg/L)
Glucose	350
Sodium acetate anhydrous	200
NH_4_Cl	300
KH_2_PO_4_	25
KNO_3_	50
CaCl_2_·2H_2_O	8
MgCl_2_·6H_2_O	12
Methylene blue (MB)	20
COD ^1^ (mg/L)	660–1025
NH_3_-N (mg/L)	83–112
pH	6.0–8.0
SS ^2^ (mg/L)	200–300

^1^ chemical oxygen demand; ^2^ suspended solids.

**Table 2:** COD, true color, and NH_3_-N in the integrated process (average values in concentration).

Process Unit	COD		True Color		NH_3_-N	
	Con. (mg/L)	Rem. (%)	Abs	Rem. (%)	Con. (mg/L)	Rem. (%)
Raw	845.6	0	0.571	0	98.4	0
IPMBR ^1^-supernatant	134.7	84.1	0.083	85.5	24.1	75.5
IPMBR effluent	62.9	92.6	0.044	92.3	15.8	84.0
CMBR ^2^-supernatant	239.0	71.7	0.178	68.8	31.3	68.2
CMBR effluent	162.1	80.8	0.144	74.8	23.8	75.7

^1^ immobilized photosynthetic bacteria MBR; ^2^ controlled MBR.

## Data Availability

Not applicable.
